# A Data Driven Model for Predicting RNA-Protein Interactions based on Gradient Boosting Machine

**DOI:** 10.1038/s41598-018-27814-2

**Published:** 2018-06-22

**Authors:** Dharm Skandh Jain, Sanket Rajan Gupte, Raviprasad Aduri

**Affiliations:** 1 Department of Computer Science and Information Systems, Birla Institute of Technology and Science Pilani, K K Birla Goa campus, Zuarinagar, South Goa, Goa, India; 2 Department of Biological Sciences, Birla Institute of Technology and Science Pilani, K K Birla Goa campus, Zuarinagar, South Goa, Goa, 403726 India; 3 Present Address: Faculty of Electronics and Information Technology, Warsaw University of Technology, Warsaw, Poland

## Abstract

RNA protein interactions (RPI) play a pivotal role in the regulation of various biological processes. Experimental validation of RPI has been time-consuming, paving the way for computational prediction methods. The major limiting factor of these methods has been the accuracy and confidence of the predictions, and our in-house experiments show that they fail to accurately predict RPI involving short RNA sequences such as TERRA RNA. Here, we present a data-driven model for RPI prediction using a gradient boosting classifier. Amino acids and nucleotides are classified based on the high-resolution structural data of RNA protein complexes. The minimum structural unit consisting of five residues is used as the descriptor. Comparative analysis of existing methods shows the consistently higher performance of our method irrespective of the length of RNA present in the RPI. The method has been successfully applied to map RPI networks involving both long noncoding RNA as well as TERRA RNA. The method is also shown to successfully predict RNA and protein hubs present in RPI networks of four different organisms. The robustness of this method will provide a way for predicting RPI networks of yet unknown interactions for both long noncoding RNA and microRNA.

## Introduction

Ribonucleic acids, RNA, have shown to be essential for a myriad of biological processes ranging from genetic information storage to being active enzymes due to their structural diversity. Many functions discovered for RNAs involve the binding of RNA binding proteins (RBPs) to specific sequences or structural motifs within the RNA. These RNA-protein interactions (RPI) govern the assembly and function of ribonucleoprotein particles (RNPs), such as ribosomes and spliceosomes, play principal roles in viral replication and translation, regulation of gene expression *via* chromatin remodeling, neuronal RNA regulatory systems, plant defense mechanisms, and biogenesis of microRNA^1–6^. At the turn of the century, the discovery that most of the human genome is transcribed into noncoding RNA (ncRNA) and is involved in regulation has led to renewed interest and a paradigm shift in the field of RPI^7–10^. Two of the most widely used techniques to determine high-resolution structures of biological molecules are X-ray crystallography and Nuclear Magnetic Resonance (NMR) spectroscopy. Even though both methods have yielded highly informative atomic resolution structures of RNA-protein complexes, the number of available structures is abysmally low compared to the protein structures. Part of this problem can be attributed to the difficulty in obtaining well-ordered RNA crystals and the complex nature of NMR spectra due to a severe overlap of proton resonances and lower proton density of RNA residues than proteins. However, recent advances in low-resolution high throughput methods have shed light on the varied atlas of these interactions. With the advent of techniques such as HITS-CLIP^11^ and PAR-CLIP^12^, there is a better understanding of the role of ncRNA in biology and the role of RPI in general, however, these experiments are often expensive and time-consuming. This has led to the development of computational tools to predict RPI.

Nowadays, a widely used computational technique in predicting bio-macromolecular interactions is Machine Learning (ML), a type of Artificial Intelligence (AI) that enables computers to learn to perform a task without programming them explicitly. The ML models can be supervised, by providing the machine with the correct output for each corresponding input or unsupervised, wherein no outputs are provided and the machine learns the relationships between the inputs, an example of which would be clustering. RPI prediction is a type of supervised learning problem, specifically, a binary classification problem in which the goal is to predict whether a given RNA-protein pair is interacting or not. The first of its kind prediction method for RPI was developed by Pancaldi and Bähler using the Random Forest (RF) and Support Vector Machine (SVM) algorithms and using several parameters (features in ML parlance) such as protein localization, genetic interactions, chromosomal localization, and predicted protein structure as inputs to the model^13^. Even though this method led the way for using computational tools for predicting RPI, it was limited due to its requirement for experimental data. Soon after, Muppirala *et al*., developed a similar method, RPISeq, but based solely on RNA and protein sequence information^14^. RPISeq is based on the curated RNA-protein interactions obtained from PRIDB^15^, a database of RNA-protein structures extracted from PDB, and used RF and SVM as the classifiers. Recently, Suresh *et al*., have proposed RPI-Pred, an SVM based prediction method utilizing protein and RNA secondary structural information as input features^16^. More recently, predicting RPIs using Deep Learning has been proposed^17^. Besides the sequence-based methods, RPI prediction methods such as catRAPID^18^ and lncPro^19^ use both sequence and structure information to predict RPI. Though all these methods have reasonable prediction accuracies on both cross-validation tests and external databases, the confidence of the predictions is very low and improvements are sought in dealing with RPI involving short RNA or protein sequences such as the ones present in the regulation of telomeres (TERRA RNA and the RBP interactome) and biogenesis of microRNA (microRNA and its interactions with RBPs).

In binary classification, the model needs to be trained on positive and negative data, i.e. examples of both interacting and non-interacting sequence pairs. All the methods mentioned above use X-ray crystallography derived structures of RPI as a positive dataset. Since experimentally verified non-interacting RNA-protein pairs are scarce, if any, a negative dataset is created by random jumbling of RNA and proteins in the positive dataset with redundancy conditions applied to eliminate false negatives. One major hurdle in doing so is the lack of confidence in the negative dataset that might bias the model’s predictions. Another major component in the successful implementation of ML algorithms is the selection of features. Usually, the RNA is encoded using a window size of four nucleotides, making a 256-dimensional vector, and the protein encoding is different in different methods but essentially follows the conjoint triad method developed for predicting protein-protein interactions (PPI) by Shen *et al*.^20^. All these methods fail to address several key observations of RPIs:(1) The amino acid classification used in these methods based on PPI work will not be relevant to the RPI prediction as the amino acid interaction propensities are different for PPI and RPI^21–23^; (2) The conjoint triad method assumes three amino acids acting as a structural unit in the interactions but in RPI the minimum structural unit required for interactions is five (see results); and (3) the presence of modified bases in RNA that play an essential role in RPI are generally neglected since they are usually labeled as X in the *fasta* files used for generating the feature vectors. For example, the crystal structure of the *T. thermophilus* aspartyl tRNA-synthase bound to the tRNA^Asp^ from *E. coli* clearly shows interactions between the Glu91 of the protein with the Queuosine, a modified guanosine base, at position 34 of the tRNA^24^. Modified residues have also been shown to provide stability to the overall structure of the tRNA which might influence the binding of proteins to the tRNA^25^.

To enhance the accuracy and confidence of predicting RPIs with sequence information alone we have developed *XRPI*, an ML-based method using the Extreme Gradient Boosting classifier, XGBoost, with features based on data-driven parameters, such as the amino acid interaction propensities and minimum structural unit (window size). Firstly, we have extracted biologically significant features from the RPI structural dataset itself. We have calculated the RPI specific amino acid interaction propensities and have scanned the entire RPI to obtain the length of the optimum binding interface. Interestingly, both the amino acid propensities as well as the optimum binding interface are different for RPI as compared to PPI. Though XGBoost has been applied in several data science challenges, its application in the field of computational biology has been limited^26^. We have optimized XGBoost to maximize its applicability and performance in dealing with biological systems. To overcome overfitting of the data, we have carefully designed a negative dataset consisting of pairs that are not similar to any known interacting pairs. Comparison with existing methods has shown that *XRPI* is the best performing method on NPInter, a database of curated RPI sourced from high throughput data and literature^27,28^, with 97.8% accuracy as well as TeloPIN, a network of RPI involved in telomere function^29^, with 99.4%. Our results indicate that *XRPI* does consistently well both in predicting RPI wherein the input sequence information is either large (NPInter) or very sparse (TeloPIN). Another advantage of *XRPI* is its computational efficiency allowing processing of large batches in seconds. We believe that the robustness of *XRPI* will greatly enhance the prediction ability of RPI and the associated RPI networks involving not only the long ncRNA but also microRNA and shorter non-coding RNA.

## Results and Discussion

*XRPI* is developed based on XGBoost, an optimized variant of the Gradient Boosting Machine, using data-driven parameters from high-resolution structures of RNA protein complexes. In this method, the amino acids are classified into four classes based on their respective interaction propensities in RPI. Each of the four monomers of RNA is considered separately along with any modified base belonging to the same class as the parent nucleotide. To account for the nearest neighbor effects, that control the structural context of the protein and RNA interactions, an interface size of five is considered for both protein and RNA.

### Datasets

As of 14^th^ May 2016, 953 RNA-protein complexes with a resolution of ≤3 Å were deposited in the Protein Data Bank^30^ (www.rcsb.org). Out of the 953 complexes, 351 complexes were rejected due to the absence of any valid RNA or protein chain (see Methods). Any RNA and protein chain in a given complex is classified as interacting if the distance between any pair of atoms of these two chains is less than 8 Å (this distance cut off was used to account for interactions involving bridging water or ion molecules)^21^. This resulted in a non-redundant dataset of 2825 unique RNA-protein interacting pairs (RPI2825). Since the RNA protein complex structures deposited in PDB are mostly ribosomal complexes, the RPI2825 is split into two parts: RPI2435 and RPI390 containing only the ribosomal RPIs and the non-ribosomal complexes respectively (Supplementary Table 1). The performance of any ML algorithm depends not only on the positive but also the negative dataset. Since there is no such negative dataset for RPI we have generated a negative dataset by random jumbling of RNA and protein chains from monomeric and multimeric RNA protein complexes. Redundancy and similarity with positive dataset conditions are applied to make sure that the negative dataset is non-overlapping and unique (see Methods).

### Feature Synthesis

**Figure 1:**
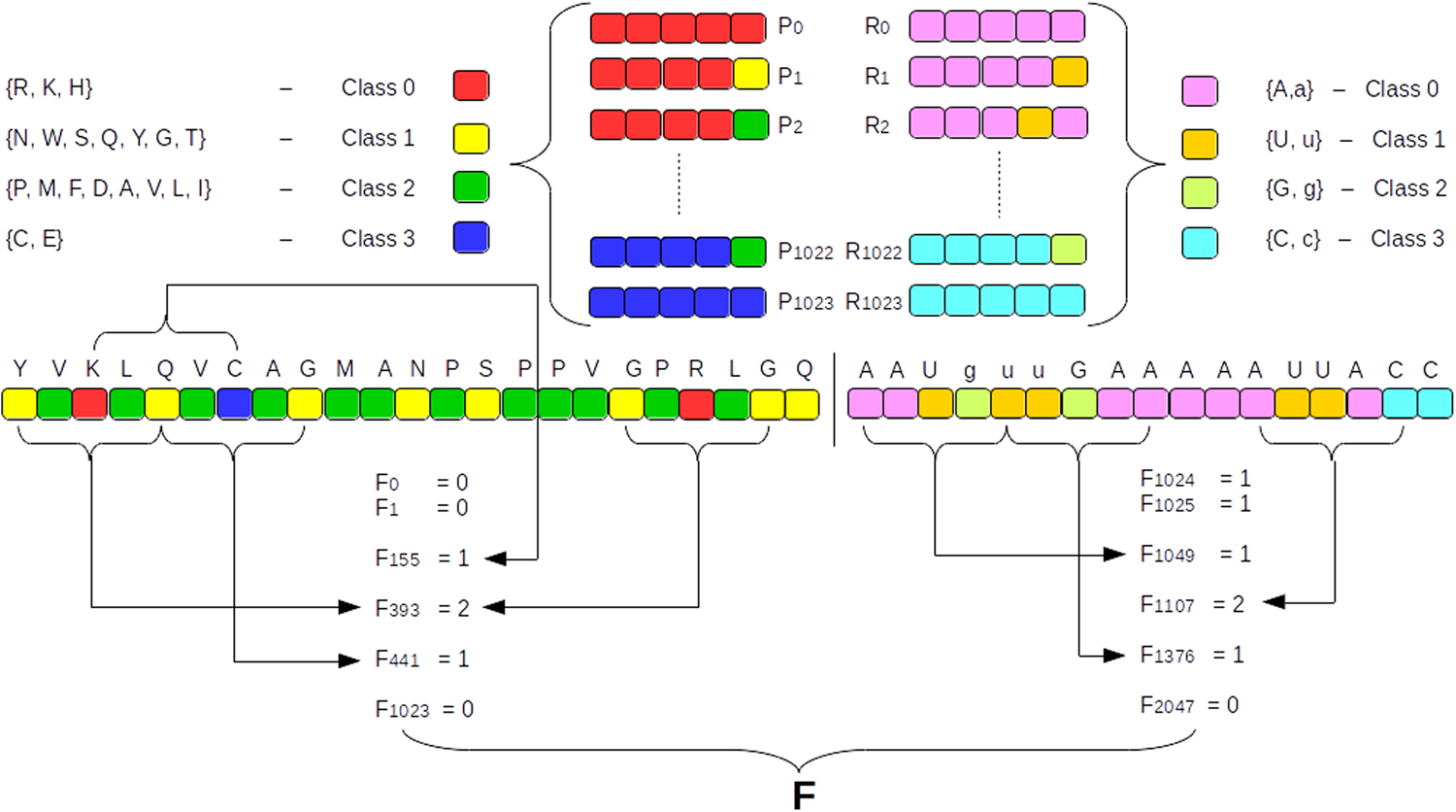
A schematic of the feature generation used in the current method. F_0_ to F_1023_ represent the protein features (see text) and F_1024_ to F_2047_ belong to RNA. These two vectors are concatenated to make the final feature vector of the RPI. The different classes are color coded for clarity. The lower cases of nucleotides refer to the modified residues corresponding to that particular nucleotide.

The features have been generated using the standard *k*-mer representation. The amino acids have been classified into four categories and the nucleotides retain their individual monomers. This classification was a result of the interaction propensity calculations performed on data obtained from experimentally determined crystal structures (see Methods). The interface size for interactions or the window size (*k*) for the feature generation is defined as a Minimal Structural Unit (MSU) equal to five. The resulting *k*-mer representations of the RNA and protein sequences are concatenated to form a single feature vector which is then fed into the XGBoost model (Fig. 1).

### Prediction Accuracy

The current prediction models are trained on RPI2825, RPI2435, and RPI390 using the amino acid and nucleotide classification described above and with the MSU as the window size. The performance of the models is evaluated using a ten-fold nested cross-validation. The prediction results for the three datasets are presented in Table 1. The averaged confusion matrix, showing the false positive and false negative values besides the true positive and true negative values is provided in Supplementary Fig. 3. The prediction accuracies are 94.3%, 95.0%, and 87.1% for the comprehensive dataset RPI2825, ribosomal dataset RPI2435, and non-ribosomal dataset RPI390 respectively.

**Table 1:** Prediction results of the test datasets (RPI2825, RPI2435, and RPI390) using ten-fold nested cross-validation.

Dataset^*^/Metric^$^	RPI2825	RPI2435	RPI390
Accuracy	0.943 (0.012)	0.950 (0.015)	0.871 (0.023)
Precision	0.953 (0.009)	0.955 (0.012)	0.891 (0.017)
Recall	0.931 (0.015)	0.949 (0.014)	0.843 (0.024)
F-Score	0.942 (0.013)	0.952 (0.016)	0.865 (0.019)
Area under ROC curve	0.975 (0.014)	0.987 (0.011)	0.914 (0.016)

^*^The dataset refers to the comprehensive (RPI2825), ribosomal (RPI2435), and nonribosomal (RPI390) datasets. Standard deviations of predictions from the ten folds are mentioned in parentheses.

^$^Please refer to the methods for definition of the metrics.

### Comparison of MSU with conjoint triad representation (a window size of five vs. three)

The existing RPI prediction methods using sequence alone information are based on the classification of amino acids (seven classes)and the sequence descriptor (conjoint triad representation) developed by Shen *et al*., for predicting PPIs. To test the effectiveness of the current amino acid classification and the MSU descriptor, we have built prediction models using Shen *et al*., and data-driven classification with MSU and conjoint triad as descriptors on the same datasets. In the cross-validation tests of the complete RPI2825 dataset as well as on the NPInter dataset, data-driven classification with MSU (*XRPI*) has done better than the Shen *et al*., method (Supplementary Table 3).

### Comparison of XGBoost with other boosting algorithms

XGBoost offers several advantages over other tree-based ensemble methods such as Random Forests, AdaBoost, and the traditional Gradient Boosted Trees, in terms of both speed and accuracy^31^. To test the validity of choosing XGBoost over other tree-based boosting algorithms we constructed prediction models using these algorithms and conducted two tests: (1) with the same parameters as the XGBoost classifier, such as the learning rate and the number of trees/iterations, wherever applicable, and (2) with each algorithm’s parameters being independently optimized. Although all the algorithms (with the exception of AdaBoost) have comparable performance on RPI2825 and NPInter, they fail to perform well on the TeloPIN dataset with the GBTree and AdaBoost performing no better than random guessing. This suggests that these boosting methods have learned patterns specific to the RPI2825 dataset and are unable to generalize their predictions to the TeloPIN dataset, consisting of a central RNA molecule made of a repetitive hexamer sequence. The comparative analysis clearly showed the advantage of XGBoost both in the cross-validation as well as on external databases (Supplementary Table 4). It is clear that in the case of biological sequence data, overfitting can happen even when model parameters are chosen through cross-validation making the traditional tree-based algorithms to fail. In contrast, XGBoost is a robust classifier that provides strong regularization methods to prevent overfitting and over-specialization which makes it an ideal candidate as a classifier for RPI prediction.

### Comparison of XRPI with existing methods

We have compared the performance of *XRPI* with the existing RPI prediction tools RPISeq^14^, RPI-Pred^17^, and lncPro^19^. For making reliable comparisons, we have trained our models on RPI2241 and RPI369 datasets provided by RPISeq (Table 2). Instead, we could have used the current RPI2825 and RPI390 datasets but that would require retraining the RPISeq and RPI-Pred models on the newer datasets. *XRPI* and RPISeq are the only methods that are based only on the sequence information; the other methods used here required obtaining secondary structural information for either or both RNA and proteins. Please refer to the Supplementary Notes for a detailed description of the comparative study. We have also compared the performances of these methods on external datasets from TeloPIN and NPInter.

**Table 2:** Comparative analysis of the performance of *XRPI*, RPISeq-RF, RPISeq-SVM, and RPI-Pred on RPI2241 and RPI369 datasets using ten-fold cross-validation.

Method	RPI2241	RPI369
*XRPI*	0.960	0.931
RPISeq-RF	0.896	0.762
RPISeq-SVM	0.871	0.728
RPI-Pred	0.84	0.92

Numbers reported are the prediction accuracies. Numbers for RPISeq and RPI-Pred are taken directly as reported from^14^ and^16^ respectively.

In our tests, we found that *XRPI* consistently performs better than any of the existing tools. In cross-validation tests on the RPI2241 and RPI369 datasets, *XRPI* performs better than all other methods. Since this evaluation needs to be done via cross validation, we could not use the stand alone lncPro software provided by the authors to do this comparison (Table 2).

In case of TeloPIN database, where the RNA sequence is a short repeat hexamer sequence, *XRPI390* (99.4%) does better than both *XRPI2825* (88.4%) and RPISeq-SVM (90.1%). In contrast, RPISeq-RF performed poorly with accuracy less than random guessing (48.6%). RPI-Pred, where structural information is incorporated in the model building, has an accuracy of only 74.3% and lncPro which also uses structural information performed very poorly (Table 3). When the long non-coding RNA containing RNA protein complexes of NPInter are considered, the performances of the models are different compared to the TeloPIN dataset (Table 4). Again *XRPI* has higher prediction accuracy than the existing methods, albeit with *XRPI2825* doing better than *XRPI390* in this case. RPISeq-RF (95.8%) outperforms RPISeq-SVM (88.4%) and RPI-Pred has an accuracy of 96.8% and able to correctly predict all RPIs in *Drosophila melanogaster* and *Saccharomyces cerevisiae*. lncPro again performs very poorly with an accuracy of 55.5%. One possible explanation for RPISeq RF and SVM models performing differently on different datasets may be due to the lack of regularization mechanisms in the Random Forest algorithm which may lead to the underperformance of these models in RPI containing sparse data as is the case with TeloPIN having a short hexamer repeat of RNA. Though RPISeq does perform well on the external datasets depending on the model (SVM vs. RF), the confidence of predictions is very low^14^. RPI-Pred performs reasonably well on NPInter dataset but the difficulty in obtaining the RNA secondary structure and protein structural blocks is a major hindrance in using this tool. On the other hand, lncPro doesn’t perform much better than random guessing in our test. Surprisingly, IPMiner^16^ fails to correctly predict any of the RPIs either in the NPInter or TeloPIN datasets (see Supplementary Notes).

**Table 3:** Comparative analysis of the performance of *XRPI*, RPISeq-RF, RPISeq-SVM, RPI-Pred, and lncPro on TeloPin dataset.

Method	Human	Mouse	Total (%)
*XRPI*-2825	124/140	36/41	160/181 (88.4%)
*XRPI*-390	136/140	40/41	176/181 (97.29%)
RPISeq-SVM	130/140	33/41	163/181 (90.1%)
RPISeq-RF	68/140	20/41	88/181 (48.6%)
RPI-Pred	104/140	31/41	135/181 (74.6%)
lncPro	9/140	1/41	10/181 (5.5%)

^$^The number of correctly predicted RPIs is shown. In parenthesis is the prediction accuracy.

**Table 4:** Comparative analysis of the performance of *XRPI*, RPISeq-RF, RPISeq-SVM, RPI-Pred, and lncPro on NPInter v3.0 golden dataset.

Method	*H. sapiens*	*M. musculus*	*D. melanogaster*	*S. cerevisiae*	Total (%)
*XRPI*-2825	1533/1560	225/237	18/19	200/204	1976/2020 (97.8%)
*XRPI*-390	1498/1560	216/237	16/19	202/204	1932/2020 (95.6%)
RPISeq-SVM	1347/1560	219/237	17/19	202/204	1785/2020 (88.4%)
RPISeq-RF	1483/1560	234/237	18/19	201/204	1936/2020 (95.8%)
RPI-Pred	1529/1560	201/237	19/19	204/204	1955/2020 (96.8%)
lncPro	784/1560	191/237	15/19	131/204	1121/2020 (55.5%)

^$^The number of correctly predicted RPIs is shown. In parenthesis is the prediction accuracy.

*XRPI* trained on RPI2825 (*XRPI*2825) is the best performing method on NPInter with 97.8% accuracy and comes in a close second on TeloPIN with 88.4%. It is the only method that does consistently well on both datasets, in spite of vastly differing RNA compositions. The lack of long RNA sequences in the RPI390 (average length of RNA in RPI390 is 44 residues) and the predominance of ribosomal RNA in the comprehensive dataset RPI2825 (with the average length of RNA being 1768) might be the possible reasons for the better performance of RPI2825 in predicting long noncoding RNA protein interactions represented in the NPInter database, whereas TeloPIN interactions involve a repeat hexamer sequence where RPI390 is expected to do better. We provide the *XRPI* with the models trained on both the RPI2825 and RPI390 datasets allowing the user to choose based on the size and nature of the RPI to be predicted.

Possible reasons for the superior performance of *XRPI* over the other methods can be attributed to: (1) the RPI specific domain knowledge used in developing the prediction models and (2) the discriminatory power (positive vs. negative RPI) of XGBoost compared to other ML tools. When we have used the PPI based domain knowledge in generating the RPI prediction models using the current datasets, we have seen lesser prediction accuracies compared to the *XRPI* (Supplementary Table 3). Furthermore, analysis of the feature discrimination utilized by *XRPI* in predicting RPIs has revealed a positive correlation (0.78) between the protein MSUs chosen by *XRPI* to the MSUs often seen at the binding interface of the RPIs. In other words, *XRPI* is learning to discriminate based on biologically relevant features (http://universe.bits-pilani.ac.in/goa/aduri/xRPI). Another plausible explanation for the better performance of *XRPI* lies in the domain-specific knowledge provided to the ML.

### Network Prediction

The high confidence dataset referred to as “golden set” of NPInter v3.0^28^ is downloaded from NPInter website (http://www.bioinfo.org/NPInter/) for the network analysis. This set of NPinter v3.0 contains all known functional ncRNA interactions, verified and curated from literature. We extracted only those entries having “ncRNA-protein binding” and wherever sequence information is available. The resulting entries were then segregated according to the source organism. The corresponding protein and RNA sequence data is downloaded from “the blast database in NPInter v3.0” section of NPInter. All the RPIs involving Xist and LincRNA from *Mus musculus* were used for creating the Xist and Linc RNA networks. Telomeric proteins interaction network (TeloPIN) database consists of data on telomeric protein interactions with telomeric repeat-containing RNA (TERRA). These interactions are downloaded from the TeloPIN database^29^ (http://songyanglab.sysu.edu.cn/telopin/). All the datasets used in the current study are available at *XRPI* webpage.

These databases provide two kinds of networks: 1. single core networks with a star topology consisting of either a single RNA interacting with multiple proteins or a single protein interacting with multiple RNA and 2. Partial mesh networks with multiple interconnected RNA and protein components.

### Single core networks with a star topology

#### TERRA interactome

**Figure 2:**
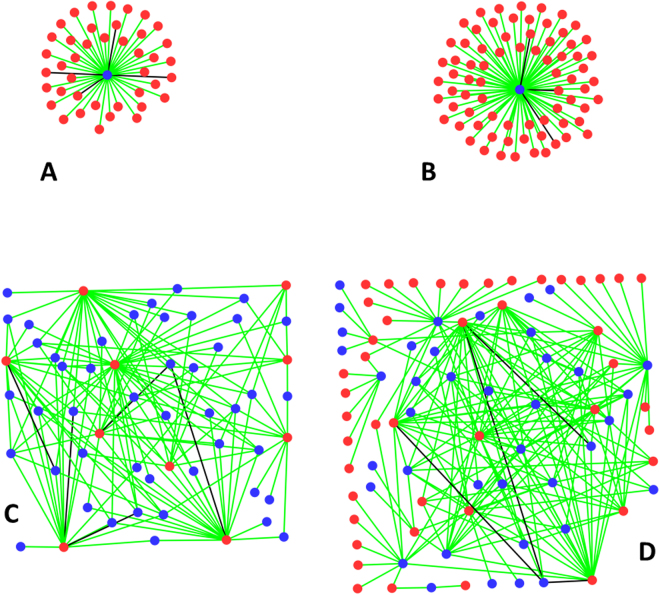
RPI networks. (**A**) TERRA RNA – protein interaction network from *Mus musculus*. (**B**) Xist RNA protein Interaction network from *Mus musculus*. (**C**) LincRNA-protein interactions network from *Mus musculus*, and (**D**) whole organism RNA protein network from *Saccharomyces cerevisiae*. RNA is depicted in blue and protein in red circles. Correctly predicted interactions are shown as green and failed predictions as black edges.

TERRA, an essential noncoding RNA (ncRNA) is involved in several regulatory processes such as telomere length control, the formation of telomeric heterochromatin, and telomere motion. TERRA performs these functions by interacting with specific proteins and recruiting them to the telomeres^32^. The TeloPIN database currently has TERRA networks of two organisms - *Homo sapiens* and *Mus musculus* which we use to construct the networks. At the heart of this star network is the repetitive TERRA sequence - UUAGGG. Our method trained using RPI2825 successfully predicted 124 out of 140 potential TERRA-protein interactions in Humans and 36 out of 41 in Mouse giving a prediction accuracy of 88.4% (Fig. 2A). Interestingly RPI390 trained model predicted all but one of the RPIs accurately (Table 3).

#### Xist interactome

One of the X-chromosomes in the mammalian females is rendered inactive/silenced to achieve dosage compensation by the expression of a long noncoding RNA referred to as X inactive specific transcript, Xist, in a process often referred to as X-chromosome inactivation (XCI)^33^. Xist recruits several RNA binding proteins in a cascade of events to perform its function. The current method is successful in predicting 71 out of 74 Xist interactions with the proteins (Fig. 2B).

### Multi-core networks with partial mesh-like topology

#### Large intergenic noncoding RNA (lincRNA) protein interactions

Large intergenic noncoding RNAs (lincRNAs) are a kind of long noncoding RNA that do not overlap protein-coding genes^8^. Owing to their controlled interactions with regulatory proteins, these RNAs have been implicated in chromosome remodeling and gene expression^34^, pluripotency and differentiation^35^, and immune response^36^. We constructed the lincRNA interactome from *M. musculus* from the lincRNA RPI derived from NPInter golden dataset. Our method successfully predicted 142 out of 147 (with an average confidence of prediction being 81%) possible interactions involving lincRNA (Fig. 2C).

### RPI at an organism level

We were able to predict the RPI networks for all four organisms in the NPInter golden dataset. Using our method, which has correctly predicted 97.8% (95.1% for RPI390 model) of the RPI present in *S. cerevisiae*, we were able to build the RPI network which revealed multiple hubs made of both proteins and RNA (Fig. 2D and Table 4). For *H. sapiens*, our method could correctly predict 1533 out of 1560 RPIs (98.3% for the RPI2825 and 95.1% for the RPI390 datasets) with confidence levels of 88%. For *D. melanogaster* and *M. musculus*, the prediction accuracies are 94.7% and 94.9% respectively (Table 4).

## Materials and Methods

### Dataset Preparation

X-ray crystal structures of RNA protein complexes with resolution ≤3 Å are downloaded from PDB databank (www.rcsb.org). A minimum length cutoff of 25 and 15 residues per chain is used for proteins and RNA respectively to be considered as a valid chain in the complex. Any RNA and protein pair in a given complex is classified as interacting if the distance between these two chains is less than 8 Å. The resulting 15088 positive RNA-protein interacting pairs are checked for redundancy, and exact pairwise duplicates are removed to obtain a non-redundant dataset of 2825 unique positive RPI pairs (RPI2825). These chains are manually curated to incorporate the modified nucleotide bases by replacing the “X” with the corresponding parent nucleotide, for example, uridine for pseudouridine, in the *fasta* sequence, which is otherwise ignored in previous studies. There are a few modified amino acid residues in protein chains and are treated in the same manner as the RNA. To overcome the bias towards the ribosomal RNA protein structures, two more datasets: RPI2435 - consisting of all the known ribosomal complexes in the RPI2825 dataset, and RPI390 - consisting of only the non-ribosomal complexes from RPI2825 are also used in generating the prediction models. The PDB IDs of the RNA protein complexes used in the current study can be downloaded from http://universe.bits-pilani.ac.in/goa/aduri/xRPI.

The negative dataset for the training is prepared as follows: The RNA protein complexes having only one RNA and one protein chain are classified as monomeric complexes and all others are considered as multimeric complexes. A large set of potential “negative” RNA-protein interaction pairs are generated from the monomeric complexes (by pairing RNA from one monomeric complex with proteins from all other monomeric complexes except, from the complex that the RNA is part of) and the multimeric complexes (here, if RNA chains R1 and R2 are interacting with protein chains P1 and P2 respectively in a multimeric complex, R1 is paired with P2 and R2 is paired with P1). For a pair in this generated set to be considered as truly negative: (1) it should not be in the positive RPI list; (2) the protein in a negative RPI must have less than 30% sequence similarity to all the proteins that the RNA of the given negative RPI is paired with in the positive RPI set (for example, if R1 and P1 are paired in the negative RPI, P1 should have less than 30% sequence similarity with all the proteins that are paired with R1 in the positive RPI set), similar check is done for the RNA; and (3) In the negative RPI set itself, all the proteins/RNA that are paired with the same RNA/protein should not have more than 30% sequence similarity. This has resulted in 7561 unique negative RNA-protein interaction pairs, of which we have selected a subset of 2825 in order to balance the positive and negative datasets for training. This subset is chosen in such a way that the ratio of unique protein chains and unique RNA chains is roughly the same in both the positive set and the negative set.

### Classification of Amino Acids and Nucleotides

It is well known that the amino acid interaction propensities for PPI are different compared to RPI and DNA-protein interactions^21,22,37^. To calculate the amino acid interaction propensities in RPI, we have defined the binding interface of an RPI using a 5 Å distance cutoff between the RNA and protein chains. Once the binding interface(s) is identified, we have calculated the RPI interaction propensity of an amino acid, to interact with the RNA as given by Equation 1.

1 $${P}_{j}\,=\,\frac{{I}_{j}/{I}_{n}}{{N}_{j}/{N}_{n}}$$

where *P*_*j*_ is the RPI interaction propensity of amino acid *j*, ${I}_{j}$ is the total number of amino acids of type *j* at the binding interface and ${I}_{n}$ is the total number of amino acids that are interacting at the binding interface in all of the RPIs in the dataset. ${N}_{j}$ is the total number of non-interacting amino acids of type *j* at the binding interface and ${N}_{n}$ is the total number of non-interacting amino acids at the binding interface in all of the RPIs in the dataset.

This resulted in formation of four distinct groups (Supplementary Table 2A). As expected positively charged amino acids (arginine, lysine, and histidine) showed the highest propensity and are grouped in one class. Negatively charged amino acids aspartic acid and glutamic acid have shown very low propensity but aspartic acid has the same propensity as the other non-polar hydrophobic amino acids proline, methionine, phenylalanine, alanine, valine, leucine, and isoleucine. Though both are negatively charged, aspartic acid shows higher propensity than glutamic acid due to interactions involving the backbone atoms rather than the side chain and the smaller side chain may provide the extra flexibility. As a result, aspartic acid is grouped along with the hydrophobic amino acids proline, methionine, phenylalanine, alanine, valine, leucine, and isoleucine. Cysteine and glutamic acid having the lowest propensity and are grouped into one class. The remaining amino acids, asparagine, tryptophan, serine, glutamine, tyrosine, glycine, and threonine make up the final group. Since each class of amino acids share similar physicochemical properties and are mostly conservative substitutions in a protein, the information content is not lost in this reductionist approach. The same analysis for RNA nucleotides showed similar propensities for all the four residues and hence a four-mer representation of the nucleotides is used. The modified residues are considered as belonging to their parent nucleotide class (Supplementary Table 2B).

### Minimum Structural Unit

The minimum structural unit of proteins and RNA is composed of five residues (the smallest structural unit of protein, for example, a beta-hairpin/turn is defined by the three residues in the apical loop/turn and the flanking residues on the N and C termini and in the case of RNA the smallest structural unit, a hairpin loop, needs to have at least three residues in the apical loop with a loop closing base pair). Besides, In case of RNA, the minimum structural unit often used in dynamic programming algorithms such as Mfold^38^ to predict RNA secondary structure is made of five residues. Based on these observations we choose a descriptor (window size) of five residues to represent both the protein and RNA sequences. This five residue window, named minimum structural unit, MSU, takes into account the effect of two adjacent nearest neighbors on each side of the central residue. This rationale is supported by the observation that the optimum binding interface in an RPI, as derived from the curated dataset obtained previously is found to be of five residues (see methods and Supplementary Fig. 2). To elucidate the MSU, we have considered any stretch of amino acids or nucleotides that are within 5 Å with their interacting partners. In deriving the MSU, we have used 5 Å cutoff instead of 8 Å to avoid any bridging water mediated interactions and specifically look at the direct amino acid interaction propensities. We have allowed a maximum of three intervening residues that are not interacting to be part of the binding interface with the bordering amino acids interacting. For example, if amino acids at position 5 and 9 are interacting with the RNA and there are no other amino acids in the protein chain that are within 5 Å distance of the RNA chain, then the optimum binding interface of the protein for this RPI is considered as five (allowing the amino acids at 6, 7, and 8, though not interacting, as part of the binding interface).

### Representation of RNA and Protein sequences

The RNA and protein sequences are represented using a *k*-mer feature representation. This representation uses a window of size n to create a histogram of frequencies for each possible n-mer. We used a window size of five, an MSU representation, for both RNA and protein chains. This technique enables us to map sequences of variable length to a finite dimensional vector whose size depends on the width of the window and the classification used. The vectors are further normalized by scaling each feature to be in the range of [0,1] by subtracting the minimum frequency from each feature’s frequency and dividing by the maximum frequency of that feature for a given RPI, in order to balance the effect of high frequencies found in longer chains. Here each protein and RNA is represented by a 1024 (4 × 4 × 4 × 4 × 4) dimensional vector. One such vector is constructed for each RNA and protein and they are appended together to create the final vector representation of the RNA-protein pair (Fig. 1).

### Machine Learning Algorithms

We used the implementation provided in the XGBoost Python library which is optimized for distributed systems. The XGBoost model was trained using 200 estimators and a learning rate (η) of 0.25 with each tree having a maximum depth of eight. The L1 regularization parameter was set using cross-validation to 1.12 and the L2 regularization parameter was set to 18.51 before training. The sub-sample ratio was set to 0.9 and the loss function to be optimized was the “binary:logistic” function, otherwise known as the log-loss function, which is well suited for binary classification tasks. The optimized XGBoost models were trained on a 16 core CPU to speed up the learning process. Parameter optimization and evaluation of the models is done using 10-fold nested cross-validation (Supplementary Notes and Supplementary Fig. 1).

### Evaluation Metrics

We have used the following metrics to assess the performance of the method.

$$\begin{array}{rcl}Accuracy & = & (TP+TN)/(TP+TN+FP+FN)\\ Precision & = & TP/(TP+FP)\\ Recall & = & TP/(TP+FN)\\ F-Score & = & 2(Precision\times Recall)/(Precision+Recall)\end{array}$$

Where, TP is true positives, TN is true negatives, FP is false positives, and FN stands for false negatives.

### Data availability

*XRPI* is freely available at http://universe.bits-pilani.ac.in/goa/aduri/xRPI. All the datasets used in the current study, as well as the comparative analysis metrics, are also available at *XRPI* website. Since user-friendly and publicly accessible web-servers represent the future direction for developing practically more useful models^39–41^, we have developed a webserver that provides *XRPI* as an online service. A standalone Linux program has also been provided so that users can process multiple RNA-protein sequence pairs efficiently on their local systems. The links for the web server as well as the binary files are freely available at the *XRPI* website http://universe.bits-pilani.ac.in/goa/aduri/xRPI.

## Conclusions and Future Work

One way to improve ML-based prediction models of biological interactions is to apply novel ML techniques and/or through domain knowledge based representation of biological information. Here, we propose ***XRPI***, an RNA protein interaction prediction tool using sequence information alone as input, based on XGBoost, a boosting ML algorithm that hasn’t been explored in biological systems extensively, and features obtained from understanding the RPI at a molecular level.

We have constructed 3 different predictive models; one is trained on only ribosomal RPIs (RPI2435), another on non-ribosomal RPIs (RPI390), and a comprehensive model that is trained on the complete dataset of 2825 RPIs (RPI2825) derived from the high-resolution x-ray crystallography structures deposited in the protein data bank. In developing *XRPI*, we elucidated RPI specific knowledge about the optimum binding interface (MSU) and RPI specific amino acid/nucleotide interaction propensities which are then used to represent the RPI to ML algorithms. The RPI specific properties elucidated in the current study will definitely expand the knowledge of RNA protein interactions in general. We believe that using domain-specific knowledge (either readily available or needs to be derived as is the case here) greatly enhances the performance of ML-based prediction models. To the best of our knowledge, *XRPI* is the first of its kind sequence alone based prediction model that uses domain-specific knowledge. The use of XGBoost algorithm that has the ability to control overfitting and over-specialization also enabled superior performance of *XRPI*. This is particularly important in the context of biological sequence data where models perform well during the cross-validation step but fail to generalize to external testing datasets. Comparative analysis of *XRPI* with the existing RPI prediction tools has revealed the superior performance of *XRPI* in predicting RNA protein complexes involving both long noncoding RNA and smaller telomere complex RNAs.

*XRPI* surpasses the current state of the art in predicting RPI by using a superior classifier whose performance is demonstrated through evaluation on external datasets. Hence *XRPI* is an accurate and robust tool that can be used for reliably predicting RPIs with a high measure of confidence, especially in cases where the sequence information is limiting (eg. telomere network). We anticipate that the method proposed will greatly aid in predicting RPI involving not only long non-coding RNA (lincRNA) but also microRNAs.

We are currently applying the data driven parameter strategy outlined here to develop models for predicting other biomolecular interactions such as DNA-protein and protein-protein interactions. We also hope to enhance the quality of predictions from *XRPI* by incorporating structural information in our feature set. Creating a structure-aware model for location specific RNA-protein interaction prediction is another research area that we are currently exploring. Incorporating additional training data from newer RPI databases such as RAID^42^ is one more approach which might improve the predictive power of *XRPI*. In the future, we plan to combine the domain knowledge and graph theoretical approaches to further the performance of *XRPI*.

## Electronic supplementary material

Supplementary Information
