# Supplementary material for: A Data Driven Model for Predicting RNA-Protein Interactions based on Gradient Boosting Machine
Source: Sci Rep. 2018 Jun 22;8:9552. doi: 10.1038/s41598-018-27814-2 (PMC6015049; doi:10.1038/s41598-018-27814-2)

**Supplementary Information**

A Data Driven Model for Predicting RNA-Protein Interactions based on Gradient Boosting Machine

Dharm Skandh Jain^1$^, Sanket Rajan Gupte^1$^, and Raviprasad Aduri^2*^

^1^Department of Computer Science and Information Systems, ^2^Department of Biological Sciences, Birla Institute of Technology and Science Pilani, K K Birla Goa campus, Zuarinagar, South Goa, Goa, India 403726

* Corresponding author: Tel: +91 832 2580 394 E-Mail: [aduri@goa.bits-pilani.ac.in](mailto:aduri@goa.bits-pilani.ac.in)

^$^ Both these authors contributed equally

**Supplementary Notes**

**XGBoost**

Prediction models based on ML often tend to perform well during the cross validation step but fail to generalize to external testing datasets. This may occur because the models learn sequence features specific to the dataset which they have been trained on, and hence perform poorly on validation sets. The main advantage that XGBoost offers over other methods that we evaluated is its ability to overcome this apparent bias by controlling overfitting and over-specialization. This is achieved by optimizing the regularization parameters. XGBoost tackles the problem of overfitting by allowing users to control the regularization parameters gamma (minimum loss reduction), alpha (L1 coefficient) and lambda (L2 coefficient) to make the model more conservative. In addition to this, XGBoost implements a variant of the tree booster called the DART (Dropout Additive Regression Tree) booster which implements dropout for trees. The dropout algorithm drops a random set of trees from the ensemble based on a dropout ratio in order to tackle the problem of over-specialization. It is an approach that is similar to Random Forests where some of the features are randomly ignored while training, but applies the concept to entire trees instead of features. But unlike RF, DART keeps adding trees to the ensemble to compensate for its deficiencies in a controlled manner, to balance tree diversity with tree specialization. The predictive power of XGBoost classifier depends mainly on the parameters that describe the model. For binary classification a good indicator of the performance of the algorithm is the logistic loss, and the two factors that play a prominent role are the learning Rate (η) and the number of iterations (n). We have observed that models with a higher learning rate required fewer iterations to reach the local minima as compared to the ones with lower learning rates. However, the models with lower learning rates had minima that were lower than those reached by models with higher learning rates. These high learning rates also led to significant over fitting after just a few iterations, which was apparent from the rise in the logistic loss values over iterations. In contrast, models with lower learning rates were more resistant to over fitting, but took longer to converge. We balanced this trade-off between the number of iterations and the learning rate by selecting values corresponding to the lowest observed log loss values, which are η = 0.25 and n = 200. A grid search was performed on a high performance computing cluster for the tree depth and the regularization parameters. We carefully chose a search space of promising parameters by experimenting on a small subset of the train data and then launched a grid search in this range of parameters. This way the search space was heuristically narrowed down and the computational power available at our disposal considerably reduced the time taken to complete the search

**Nested cross-validation**

Cross-validation is typically used to estimate the performance of a model, and not to tune its parameters. Optimization using CV gives an overly optimistic estimate of performance (*optimistic bias*) on independent test data as the parameters are iteratively refined based on the performance on the test splits, which yields a configuration that might implicitly overfit to the test splits. This may significantly affect the performance of the model on unseen test data. To perform both parameter optimization, and get unbiased estimates of the model’s generalization ability on unseen data, one has to perform k-fold nested cross-validation. In this procedure, we make k train-test splits of the dataset (called the outer splits) similar to the process followed in ordinary cross-validation. For each outer split, we use the train set to perform parameter optimization by having *k* inner splits for this set, a nested procedure from which this technique derives its name. Finally, the best performing set of parameters on the inner splits is used to train a model on the train set and apply it to the test set for each outer fold.

This nested procedure results in k different models, each having possibly different parameters (as each one was optimized on a slightly different set of the data). To create a unified prediction model for unseen data, an ensemble of these k models can be used to average all the predictions, or stacked generalization can be used with a linear model to get a weighted average of the model predictions. However, these techniques are applied when the k models are unstable, *i.e.* the performance on each fold varies significantly. In our case, the models we generated were stable and had nearly identical parameters, and similar performance on all folds. To create a final predictor, we retrained a single model with these stable parameters on the entire set, and used it for prediction on the unseen test sets (NPInter, TeloPIN etc.).

**Method comparison**

RPISeq is trained on 2241 pairs extracted from PDB and uses normalized 3-mer (conjoint triad representation) and 4-mer frequencies to represent protein and RNA sequences respectively. The amino acids classification follows the Shen *et al*., the methodology developed for PPI prediction. The two versions of RPISeq rely upon two different ML algorithms, Random Forests (RPISeq-RF) and Support Vector Machines (RPISeq-SVM) to make predictions. RPI-Pred uses higher order structural information in the form of protein blocks and five classes of RNA secondary structures along with sequence information to create an SVM based classifier using 1807 interacting training pairs as the positive dataset. In cases where structural information is not available, predicted RNA and protein structures are used. Whereas, lncPro employs various features derived from structural information of the RNA and protein (for example Hydrogen bonding and Vander Waal's interactions) to build a linear discriminant model to score each RNA-protein pair. The model is trained using 726 pairs extracted from PDB which have sequences longer than 100 bases of RNA.

*RPISeq*: RPISeq web server (<http://pridb.gdcb.iastate.edu/RPISeq/>) is used to score the sequences in hold out datasets (NPInter and TeloPIN).

*RPI-Pred*: RPI-Pred server (<http://ctsb.is.wfubmc.edu/projects/rpi-pred/>) is used to score the sequences. RPI-Pred requires predicted protein 3D structure in the form of protein blocks. To generate these predictions, we used the same prediction tool (PB-kPRED, available at <http://www.bo-protscience.fr/kpred/> as the authors. The structure predictions were calculated using the Majority Rule Method of PB-kPRED.

*lncPro*: We downloaded the lncPro software from <http://bioinfo.bjmu.edu.cn/lncpro/> and installed the predator and RNAsubopt packages which it depends on. Due to the limitations of the software, there were 40 instances in which memory corruption issues caused no score to be returned. In these cases, a score of 0 was assigned manually. The RNA folding algorithms in RNAsubopt also had overflow errors in some cases, which might have affected the score. Scores returned by lncPro were not consistent across multiple runs, ie. the same RNA-protein pair would receive slightly different scores when scored repeatedly with lncPro.

*IPMiner*:: We downloaded the source code of IPMiner from <https://github.com/xypan1232/IPMiner>. IPMiner has an option to score RNA-protein pairs that are input as separate *fasta* files (one for RNA and one for proteins). It automatically makes all possible RNA-Protein pairs and prints the interaction score. Using this feature it was easy to score the TeloPIN dataset, as a single RNA interacts with multiple proteins. However in the case of NPInter, we could not provide the inputs in this format since we do not wish to score all possible RNA-protein pairs, just the ones in the golden set. We tried to provide the inputs to the model pair by pair but this turned out to be impractical because IPMiner does not have any pre-trained models. Rather, a model is trained from scratch every time an input is given. Since this is a complex ensemble of models, it’s not feasible to do this for each of the 2020 pairs in NPInter due to the computational costs involved. We made a few minor modifications to the source code to allow us to feed a set of sequence pairs directly to the model, so that it would train once and then make a prediction for the entire batch. We did not make other changes to the model or training / prediction procedure, we only modified a small section of the data loading code to accommodate our use case. It is important to note that since a new model is trained every time, random weight initialization of the deep network means that the prediction results will be slightly different with each run. Nevertheless, we ran the code multiple times and IPMiner consistently failed to correctly identify even one of the sequences in our held out test sets (NPInter and TeloPIN) as an interacting pair.

**Supplementary Table 1.**The dataset composition

| Complexes from PDB | 953 |  |  |
| --- | --- | --- | --- |
| Curated complexes^$^ | 602 | Unique RPIs | 2825 |
| Ribosomal Complexes* | 202 | Unique Ribosomal RPIs | 2435 |
| Non-ribosomal Complexes* | 400 | Unique Non-ribosomal RPIs | 390 |
| Unique Protein chains | 899 | Unique RNA chains | 460 |

^$^ 351 complexes were rejected due to absence of valid RNA chains

* The complexes are from the 602 curated complexes

**Supplementary Table 2A.**The interaction propensities of amino acids as calculated based on equation 1. This resulted in 20 amino acids being classified into four different classes (first column). The cut off boundaries for classification using propensity values are kept at 1.5, 1.0 and 0.5. The amino acids constituting each group share similar physicochemical properties as expected.

| Class representation | Amino Acid | Propensity |
| --- | --- | --- |
| 0 | R (ARG) | 2.730 |
| 0 | K (LYS) | 2.100 |
| 0 | H (HIS) | 1.922 |
| 1 | N (ASN) | 1.424 |
| 1 | W (TRP) | 1.374 |
| 1 | S (SER) | 1.306 |
| 1 | Q (GLN) | 1.273 |
| 1 | Y (TYR) | 1.235 |
| 1 | G (GLY) | 1.232 |
| 1 | T (THR) | 1.184 |
| 2 | P (PRO) | 0.980 |
| 2 | M (MET) | 0.671 |
| 2 | F (PHE) | 0.671 |
| 2 | D (ASP) | 0.656 |
| 2 | A (ALA) | 0.623 |
| 2 | V (VAL) | 0.539 |
| 2 | L (LEU) | 0.504 |
| 2 | I (ILE) | 0.502 |
| 3 | C (CYS) | 0.488 |
| 3 | E (GLU) | 0.435 |

**Supplementary Table 2B.**The interaction propensities of nucleotides, shown as an histogram, based on equation 1. All the four nucleotides have similar interaction propensities.

**
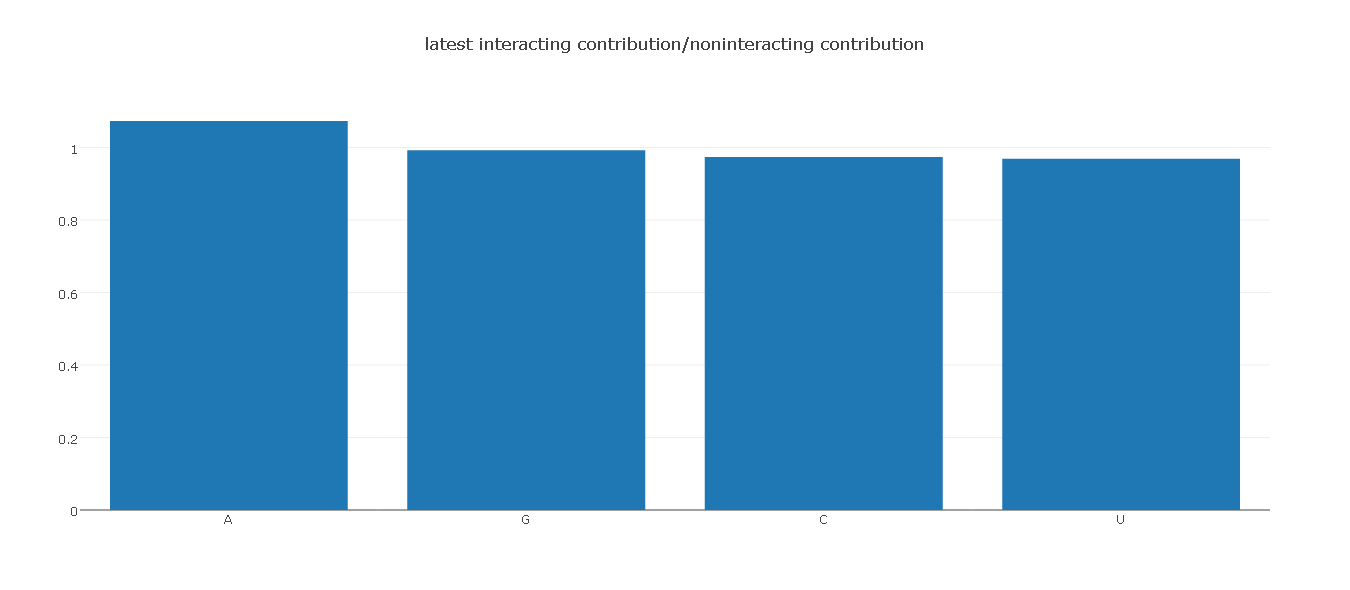
**

**Supplementary Table 3.**Network Prediction Results with varying classification and window size

1. **Using RPI2825 trained model**

| Dataset | Shen*et al* Classification  Conjoint Triad \| MSU | Data Driven Classification  Conjoint Triad \| MSU |
| --- | --- | --- |
| RPI2825 (CV) | 0.938 \| 0.939 | 0.935 \| 0.943 |
| TeloPIN - Human | 0.893 \| 0.771 | 0.807 \| 0.886 |
| TeloPIN - Mouse | 0.780 \| 0.780 | 0.732 \| 0.878 |
| NPInter | 0.973 \| 0.878 | 0.988 \| 0.978 |

1. **Using RPI390 trained models**

| Data Driven Classification  Conjoined Triad \| MSU | Shen et al Classification  Conjoint Triad \| MSU | Dataset |
| --- | --- | --- |
| 0.844 \| 0.871 | 0.861 \| 0.841 | RPI390 (nCV) |
| 0.994 \| 0.994 | 0.993 \| 0.979 | TeloPIN - Human |
| 0.951 \| 1.000 | 1.000 \| 0. 951 | TeloPIN - Mouse |
| 0.881 \| 0.951 | 0.925 \| 0.505 | NPInter |

nCV refers to the results obtained from nested cross validation

**Supplementary Table 4.**Network Prediction Results with different classifiers

1. **Using RPI2825 trained model**

| Dataset | XGBoost | AdaBoost | Gradient Boosting Tree | Random Forest |
| --- | --- | --- | --- | --- |
| *RPI2825 (nCV)* | 0.943 | 0.822 | 0.936 | 0.935 |
| Telopin– Human | 0.886 | 0.50 | 0.55 | 0.700 |
| Telopin– Mouse | 0.878 | 0.53 | 0.488 | 0.390 |
| NPInter | 0.978 | 0.960 | 0.945 | 0.930 |

1. **Using RPI390 trained models**

| Dataset | XGBoost | AdaBoost | Gradient Boosting Tree | Random Forest |
| --- | --- | --- | --- | --- |
| *RPI390 (nCV)* | 0.871 | 0.721 | 0.853 | 0.862 |
| Telopin– Human | 0.971 | 0.593 | 0.907 | 0.957 |
| Telopin– Mouse | 0.976 | 0.707 | 0.951 | 0.951 |
| NPInter | 0.956 | 0.811 | 0.911 | 0.954 |

**Supplementary Figure1.** Optimization of the XGBoost parameters: learning rate and logistic loss, as a function of the number of iterations. The “1-logistic loss” is shown with a pseudo color gradient. The optimum values for the number of iterations and learning rate correspond to the lowest observed log loss values(here it is 0.18).


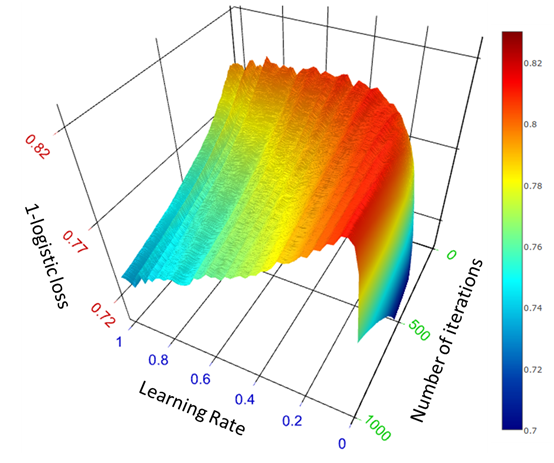


**Supplementary Figure2**. The optimum binding interface length of proteins (top) and RNA (bottom) participating in an RPI (refer to the main text for an explanation of the methodology used in calculating the binding interfaces). The length of the binding interface is on the x-axis and number of occurrences of the same in the entire RPI dataset (RPI2825) is on the Y-axis. Notice that there can be more than one binding interface present in a given RPI.

**
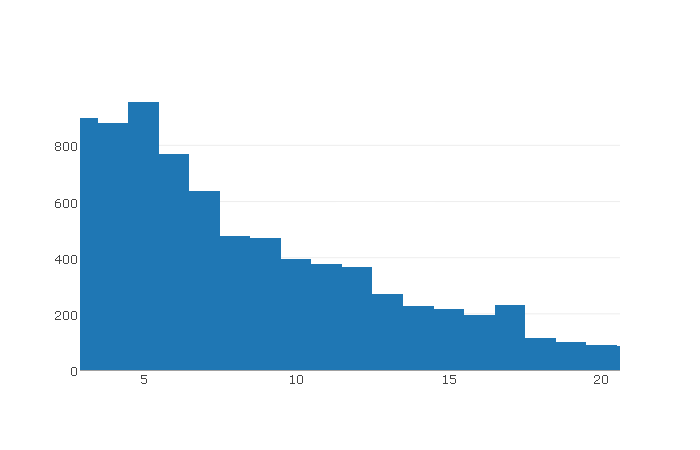
**

**
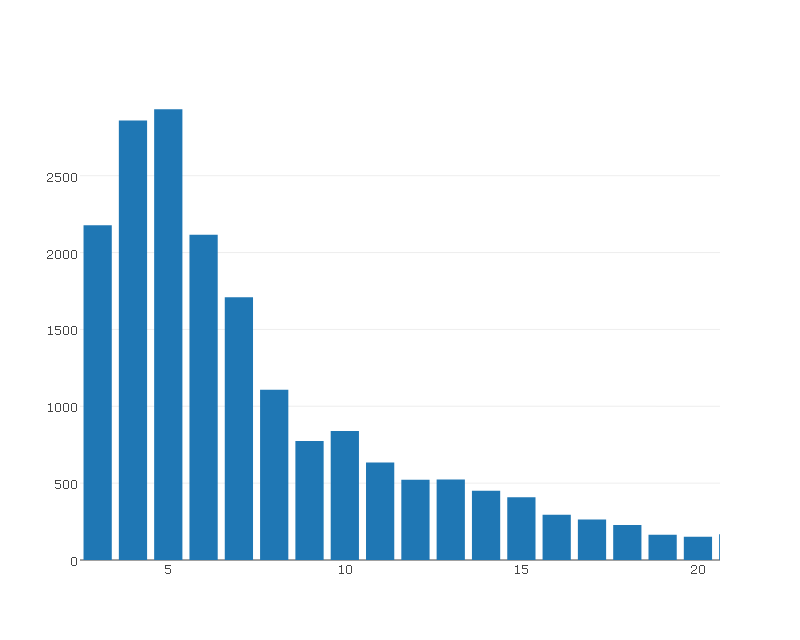
**

**Supplementary Figure3**. The averaged confusion matrix of the nested cross validation results for *XRPI* trained on RPI2825 (top) and RPI390 (bottom) datasets.


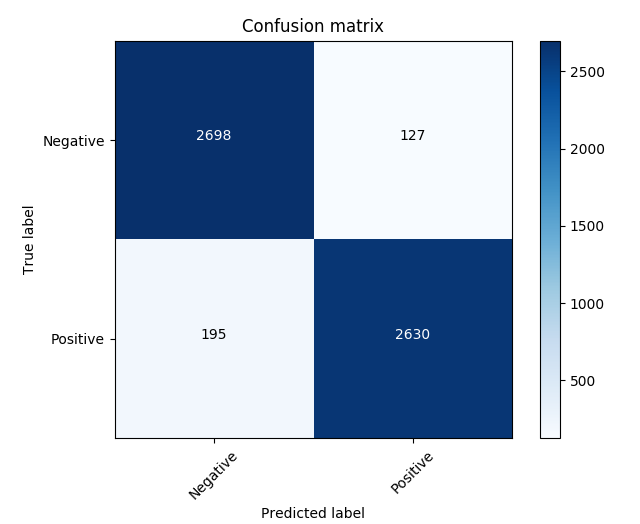


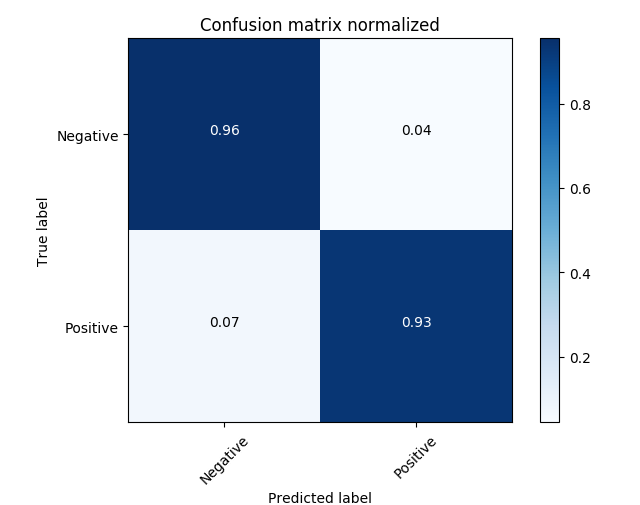

Supplement: Supplementary file 1 — Supplementary Information [file 41598_2018_27814_MOESM1_ESM.docx]
